# Combined neuroendocrine carcinoma and adenocarcinoma in the stomach: A case report

**DOI:** 10.3892/ol.2014.1825

**Published:** 2014-01-24

**Authors:** SHOUZHEN LI, XUEYUAN CAO, CHENGYI JIANG, QUAN WANG

**Affiliations:** Department of Gastrointestinal Surgery, First Bethune Hospital of Jilin University, Changchun, Jilin 130021, P.R. China

**Keywords:** neuroendocrine carcinoma, adenocarcinoma, stomach

## Abstract

Neuroendocrine carcinoma and adenocarcinoma existing in the stomach simultaneously is extremely rare. This report presents a 65-year-old male patient who was diagnosed with three types of malignant tumors in the stomach, neuroendocrine carcinoma (NEC), moderately differentiated adenocarcinoma and mucinous adenocarcinoma. In addition, the NEC and moderately differentiated adenocarcinoma existed in the same lesion and, therefore, was referred to as a mixed adenocarcinoma - NEC tumor. The patient underwent laparoscopic-assisted D2 radical total gastrectomy, Roux-en-Y esophagus-jejunum anastomosis and received FOLFOX chemotherapy for six cycles 3 weeks after surgery. Follow-up determined that the patient survived and was tumor-free 12 months after surgery. In conclusion, radical surgery combined with chemotherapy can effectively improve the prognosis of patients with these three specific tumor types simultaneously in the stomach.

## Introduction

Neuroendocrine tumors were first described by Langhans in 1867 (1). Neuroendocrine carcinoma (NEC) is a type of malignant tumor in which cells often show amine precursor uptake and decarboxylation to synthesize and secrete amine and polypeptide hormones. Gastric neuroendocrine tumors (gastric carcinoids) have been classified on the basis of pathogenesis and histomorphologic characteristics into three types differing in biological behavior and prognosis. Gastric neuroendocrine tumor types 1 and 2 are usually considered benign with a low risk of malignancy. Type 3 gastric neuroendocrine tumors are composed of different endocrine cells, including poorly differentiated endocrine and exocrine cells, which grow sporadically, irrespective of gastrin in an otherwise normal mucosa. The majority of these tumors show a low- to high-grade malignant transformation, already metastasizing at the time of diagnosis Endocrine tumors, which primarily exist in the stomach, account for only 2–6% of gastrointestinal endocrine tumors. Among all gastric cancer types, endocrine tumors account for only 0.1–0.9% of cases (2). This report presents a rare clinical case of three types of malignant tumors localized in the stomach; NEC, moderately differentiated adenocarcinoma and mucinous adenocarcinoma. Written informed consent was obtained from the patient for publication of this case report and any accompanying images.

## Case report

### Case presentation

**Figure 1:**
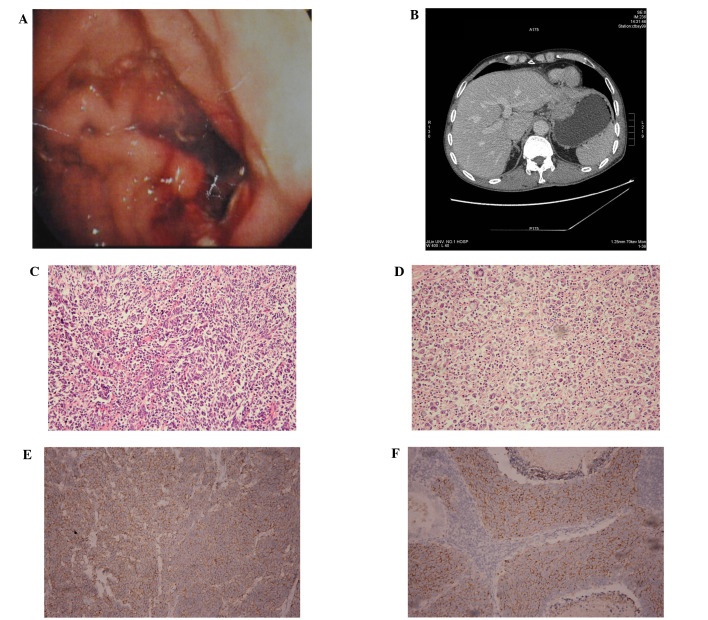
(A) Gastric fiberscopy of the tumor (neuroendocrine carcinoma and moderately differentiated adenocarcinoma) occupying the bottom of the stomach and its body curvature side. (B) Contrast-enhanced computed tomography scanning of the tumor (neuroendocrine carcinoma and moderately differentiated adenocarcinoma). (C) Neuroendocrine carcinoma with moderately differentiated adenocarcinoma and (D) mucinous adenocarcinoma were identified by microscopy (hematoxylin and eosin staining; magnification, ×100). Postoperative immunohistochemistry results confirmed the diagnosis of neuroendocrine carcinoma, by the presence of (E) synaptophysin (+) and (F) chromogranin A (+).

A 65-year-old male patient presented with occasional shortness of breath following activities and appeared weak 1 month ago, with no evidence of underlying disease. The patient had no history of lung disease and was negative for family hereditary disease. Auxiliary examination consisted of contrast-enhanced computed tomography (CT) of the stomach, which identified the following: Lesser curvature of the stomach, obvious thickening of the stomach angle, discontinuous mucous membrane layer and a coarse serous membrane layer, part of which was not clearly divided from the left side of the liver. Enhancement scanning showed uneven obvious strengthening of the tumor in the lesser curvature of the stomach. CT results identified that the staging of the tumor was T3N3M0 (Fig. 1). Gastric fiberscopy revealed that the tumor occupied the bottom of the stomach and the diameter of its body curvature side was 1.5×1.5 cm, with central depression and covered with ulcer and necrotic tissues. Additionally, the mucosal lining was rough. These results showed obvious congestion and edema. The elasticity of the inspected tissue was stiff and hemorrhaged when touched. The tumor lied in the lesser curvature of the lower part of the stomach and the body of the stomach (Fig. 1A). Biopsy of the stomach tissue specimens identified that several glands exhibited severe dysplasia, not excluding well-differentiated adenocarcinoma; therefore, advanced examination was suggested. Laboratory examination showed anemia (hemoglobin level, 71 g/l). The level of the tumor marker, CA72-4, in the blood was 9.8 U/ml (normal range, 0–6.9 U/ml) and the carcinoembryonic antigen level was 6.52 ng/ml (normal range, <3.4 ng/ml).

### Surgery

The patient underwent a laparoscopic total gastrectomy (Roux-en-Y anastomosis) under general anesthetic (D2 and R0 resection). During surgery two tumors were identified: Tumor 1 was located in the gastric curvature near the cardia, which was ~5×3.5×1 cm in size; tumor 2 was located in the back wall of the gastric body and was ~8×4.5×2 cm in size. The two tumors were 5 cm apart.

### Pathological analysis

Postoperative pathological diagnosis found that there were two independent and different morphological lesions in the stomach. Tumor 1 included two types of carcinoma (Fig. 1C), NEC and moderately differentiated adenocarcinoma, which can be referred to as mixed carcinoma or collision carcinoma, invading the subserosal connective tissue. Immunohistochemical results for NEC revealed: Chromogranin A (CgA; +) (Fig. 1E), synaptophysin (Syn; +) (Fig. 1F), vimentin (+), thyroid transcription factor-1 (+), CD117 (+) and Ki67 (+ 80%); while those for moderately differentiated adenocarcinoma were as follows: Ki67 (20%), epithelial membrane antigen (EMA; +), CK20 (+), CK7 (+) and villin (+).

Tumor 2 was mucinous adenocarcinoma invading the plasma membrane. The majority of cancer tissues were diffused by individual cells, which were clumped together to create a mass tumor, and only a few were physaliphorous cells. The vessel and nerve were infiltrated with carcinoma. Immunohistochemical analysis of the mucinous adenocarcinoma revealed: CK (+), EMA (+), Ki67 (+80 %) and vimentin (+). In the lesser curvature of the stomach, the number of the metastasis lymph nodes was 2 out of 5 total lymph nodes. All the metastasis lymph nodes were neuroendocrine carcinoma.

### Chemotherapy

The patient received six cycles of FOLFOX chemotherapy regimen 3 weeks after surgery. Follow-up revealed that the patient survived and was tumor-free 12 months after surgery.

## Discussion

The origin of mixed gland - NEC has not been fully elucidated. The majority of scholars propose that NEC in the digestive system originated in the neuroendocrine cells of the digestive system, which were derived from the endoderm and, therefore, have the same origin as gastrointestinal epithelium. Both tumor types originate from totipotent stem cells. Under the action of carcinogenic factors, totipotent stem cells undergo malignant differentiation to produce NEC, squamous cell carcinoma and adenocarcinoma. NEC and adenocarcinoma have the same risk factors, including dietary, hereditary, environmental and psychological factors. Gastric neuroendocrine tumors can be divided into three types of tumors according to the World Health Organization (WHO, 2000): Well-differentiated neuroendocrine tumor (carcinoid), well-differentiated NEC and poorly differentiated NEC (high-grade NEC; WHO, 2010) (3). The differentiation of gastric neuroendocrine tumors has been reported as a step-by-step process: i) gastric endocrine cell hyperplasia; ii) dysplasia; iii) gastric carcinoid (well-differentiated); iv) atypical carcinoid (intermediately differentiated); and v) NEC (poorly differentiated) (4). Gastric tumors have been mainly detected by gastric endoscopy (5). NEC and adenocarcinoma are difficult to diagnose and identify by gastroscopy or ultrasound. Therefore, clarifying a diagnosis can rely on histopathological examination, and immunohistochemistry is the most common method. The widely used indicators for immunohistochemistry are QrA, Syn and neuron-specific enolase, while CgA and Syn have high specificity (6,7). MEN1 gene detection from blood samples and positron emission tomography CT scanning have been reported as advanced methods for the diagnosis of NEC and adenocarcinoma. The treatment of NEC includes surgery, radiotherapy, systemic chemotherapy, biological therapy and targeted drug therapy (8).

The present report demonstrated a case of three types of malignant tumors localized in the stomach, including NEC, moderately differentiated adenocarcinoma and mucinous adenocarcinoma. NEC and moderately differentiated adenocarcinoma existed simultaneously in the same lesion, and the cells were intermixed. To the best of our knowledge, this was the first case report of three types of malignant tumors existing in the stomach at the same time. According to the grading standard of the European Neuroendocrine Tumor Association (9), gastric NEC in this case was T2N1M0, stage IIIB stomach cancer; and the mucinous gastric carcinoma was T4aN3M0, stage IV stomach cancer. The three types of malignant tumors, including NEC, existing in one stomach, were highly malignant and the patient had a poor prognosis. The patient underwent laparoscopic-assisted D2 radical total gastrectomy and Roux-en-Y esophagus-jejunum anastomosis. The surgery was successful. The patient also received FOLFOX chemotherapy for six cycles 3 weeks after surgery. Follow-up determined that the patient survived and was tumor-free 12 months after surgery. According to this case report, radical surgery combined with chemotherapy can effectively improve the prognosis of patients with these three specific tumor types simultaneously in the stomach.
